# Efficacy of artemisinin–lumefantrine for treatment of uncomplicated malaria after more than a decade of its use in Kenya

**DOI:** 10.1017/S0950268820003167

**Published:** 2021-01-05

**Authors:** Gabriel Kishoyian, Eliud N. M. Njagi, George O. Orinda, Francis T. Kimani, Kevin Thiongo, Damaris Matoke-Muhia

**Affiliations:** 1 Department of Medical Laboratory Sciences, Kenya Medical Training College, P.O. Box2268-40100, Kisumu, Kenya; 2 Department of Biochemistry and Biotechnology, Kenyatta University, P.O.BOX 43844-00100, Nairobi, Kenya; 3 Centre for Biotechnology Research and Development, Kenya Medical Research Institute, P.O. Box 54840-00200, Nairobi, Kenya

**Keywords:** Artemether–lumefantrine, efficacy, falciparum malaria, Kenya

## Abstract

**Background**

The resistance of *Plasmodium falciparum* to antimalarial drugs remains a major impairment in the treatment and eradication of malaria globally. Following the introduction of artemisinin-based combination therapy (ACT), there have been reports of delayed parasite clearance. In Kenya, artemether–lumefantrine (AL) is the recommended first-line treatment of uncomplicated malaria. This study sought to assess the efficacy of AL after a decade of use as the preferred method of managing malarial infections in Kenya. We assessed clinical and parasitological responses of children under 5 years between May and November 2015 in Chulaimbo sub-County, Kisumu, Kenya. Patients aged between 6 and 60 months with uncomplicated *P. falciparum* mono-infection, confirmed through microscopy, were enrolled in the study. The patients were admitted at the facility for 3 days, treated with a standard dose of AL, and then put under observation for the next 28 days for the assessment of clinical and parasitological responses. Of the 90 patients enrolled, 14 were lost to follow-up while 76 were followed through to the end of the study period. Seventy-five patients (98.7%) cleared the parasitaemia within a period of 48 h while one patient (1.3%) cleared on day 3. There was 100% adequate clinical and parasitological response. All the patients cleared the parasites on day 3 and there were no re-infections observed during the stated follow-up period. This study, therefore, concludes that AL is highly efficacious in clearing *P. falciparum* parasites in children aged ≥6 and ≤60 months. The study, however, underscores the need for continued monitoring of the drug to forestall both gradual ineffectiveness and possible resistance to the drug in all target users.

## Introduction

In spite of the tremendous decline of the burden of malaria over the past decade, the disease still remains a major public health concern globally [1]. Sub-Saharan Africa is reported to bear the greatest burden [1]. In 2018, for instance, approximately 228 million malaria cases and 405 000 deaths were reported worldwide. Of this, 92% and 93% of the reported cases and deaths, respectively, were from sub-Saharan Africa, with children under 5 years of age and expectant mothers being the most affected [1]. There are five species of malaria parasites that are known to infect human beings: *P. falciparum*, *P. ovale*, *P. malaria*, *P. vivax* and *P*. *knowlesi*, with *P. falciparum* being the most severe and the leading cause of morbidity and mortality worldwide [2].

The World Health Organization Global Malaria Program (WHO/GMP) recommends three key interventions for controlling and managing the effects and the spread of malaria, these include (1) prompt diagnosis and treatment with effective medicines, (2) distribution of insecticide-treated nets to attain full protection of populations at the risk of contracting malaria, and (3) indoor residual spraying as a key means to reduce and eradicate malaria transmission [2, 3]. Since the initiation of the Roll Back Malaria (RBM) project over a decade ago, there has been an increase in the distribution of long-lasting insecticide-treated nets and intense case management in most countries where malaria is endemic with reports indicating their contribution to the decline of malaria infections [4, 5]. Proper diagnosis and prompt treatment of malaria cases with effective and efficacious antimalarial drugs remains one of the cornerstones for malaria control [1]. However, resistance of the parasite to artemisinin-based combination therapies (ACTs) threatens the effectiveness and efficacy of these drugs.

*In vivo* clinical trials are among the methods used to assess antimalarial drug efficacy [6]. During these trials, patients are treated and followed up for a period of 28 or 42 days as per the World Health Organization (WHO) guidelines. WHO classifies responses to treatment as follows: early treatment failure (ETF), late clinical failure (LCF), late parasitological failure (LPF) and adequate clinical and parasitological response (ACPR) [2, 6]. *In vitro* assays are measured through malaria parasites susceptibility in culture and observation of drug concentration in which 50% of the parasite growth is inhibited (IC50) compared to the unexposed control [7].

Artemisinin resistance in drug-selected *P. falciparum* lines has been associated with decreased susceptibility of ring-stage parasites and, in some cell lines, mature trophozoites-stage parasites as well. However, an *in vitro* assay (ring-stage survival assay) study in Cambodia observed that the susceptibility of 0–12 h post-invasion rings to a pharmacologically relevant exposure to dihydroartemisinin; there were 17 times higher survival rates of culture-adapted parasite isolates [8]. Further, surveillance of drug resistance molecular markers has also been used in efficacy testing [6]. Several genetic markers associated with resistance to anti-malarial drugs have been identified. This includes mutations in the kelch propeller domain of the *k13* gene which has been associated with artemisinin resistance. Those that have been coupled with artemisinin resistance include five markers which have been validated using *in vivo* and *in vitro* assays and eight others implicated through correlation with delayed parasite clearance of k13 mutations [6].

Chloroquine (CQ) was developed in the 1930s as a more effective replacement for quinine. It was then accepted as the drug of choice for combating malaria in all disease-endemic regions [9]. CQ, which is significantly disseminated in the tissues, has a long half-life of approximately 28 days, hence highly effective [9]. The first case of *P. falciparum* resistance to CQ was reported in Thai–Cambodia border in Southeast Asia and also in South America in the 1950s. Thereafter, more cases of resistance were observed in most malaria-endemic regions, with the first cases in Africa being reported in the 1970s [10]. Malawi was the first country in the continent of Africa to cease the administration of CQ as the drug of choice for treating malaria in the year 1993 [11]. In Tanzania, CQ was used as a first-line malaria treatment drug since the 1970s, but due to its significant resistance levels, it was replaced with sulfadoxine-pyrimethamine (SP) in the year 2001. The aforementioned cases of resistance may then be said to have precipitated the emergence of artemether–lumefantrine (AL) in 2006 [11].

In Kenya, CQ-resistant *P. falciparum* was first reported in 1977; however, by 1998, resistant levels had reached 70% [12]. Like other sub-Saharan countries, Kenya replaced CQ with SP in 1999 as the official first-line in the treatment of uncomplicated malaria [13]. Thereafter, in 2004, AL was introduced after reports of SP resistance in Kenya especially at the coastal region [14, 15]. AL was made available in government hospitals in 2006 [11]. Based on the data from routine health information systems, malaria remains a major burden to the public and accounts for an estimated 19% of outpatient consultations in Kenya [16, 17]. In Kenya, there are an estimated 3.5 million new clinical cases and 10 700 deaths each year, with those in western Kenya being at a higher risk of malaria [https://www.cdc.gov/malaria/malaria_worldwide/cdc_activities/kenya.html#:~:text=In%20Kenya%2C%20there%20are%20an,of%20Health%20to%20fight%20malaria]. Pregnant women and children are at a higher risk, more so school-going children aged between 5 and 15 years old.

The Kenya national malaria control guidelines recommended dihydroartemisinin–piperaquine (DHA/PPQ) as the second-line treatment for uncomplicated malaria in the country. Usually, parenteral artesunate is the drug of choice for severe malaria; however, when unavailable, parenteral quinine is given as an alternative. During the period of transition, both ACTs and SP drugs were the drugs of choice [18]. The proportion of ACT usage increased from zero in 2004 to 48% and 69% in 2010 and 2016, respectively, while SP drug usage declined from 88% in 2004 to 39% in 2010 and 27% in 2016, respectively [18].

In spite of the successes in uptake, the curative effect of ACT, as with most other drugs, has tended to decline gradually, with studies showing a slowing action of parasite clearance in *P. falciparum*-positive patients, especially in Southeast Asia [19, 20]. Due to the looming drug resistance to AL, WHO recommended regular surveillance to monitor the performance of antimalarial drugs in use in all malaria-endemic countries [2, 6]. Therefore, this formed the background of this study, with the primary objective of assessing clinical and parasitological responses following the 3-day treatment of uncomplicated *P. falciparum* malaria with AL as stipulated in the WHO protocol.

## Materials and methods

### Study area

**Fig. 1.**
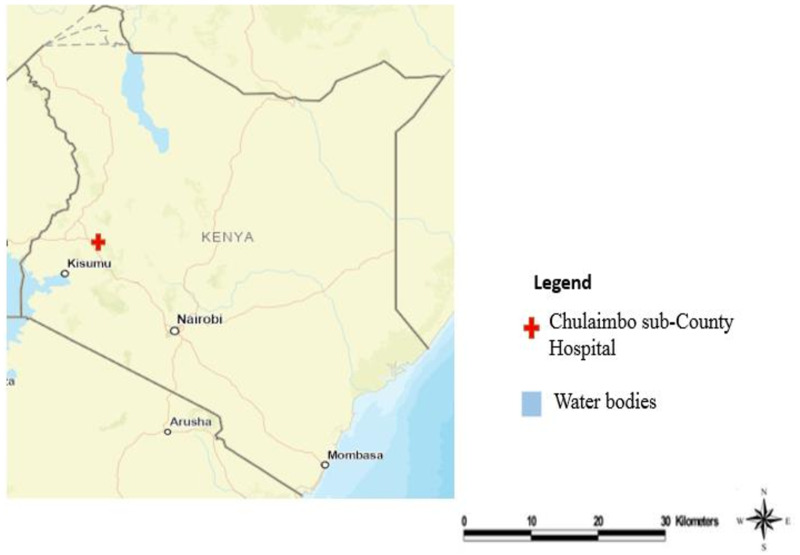
A map showing the location of Chulaimbo Sub-County Hospital, Kisumu County, Western Kenya.

We conducted the trial in Chulaimbo Sub-County Hospital, Lake Region of Kisumu County in Western Kenya (Fig. 1). The area altitude is 1131 m above sea level with yearly rainfall between 1200 and 1300 mm. Humidity ranges between 50% and 68% with temperatures ranging between 20 and 35 °C. Chulaimbo Sub-County is a malaria-endemic zone with stable *P. falciparum* transmission.

### Study population

Children aged between 6 and 60 months who visited the outpatient clinic at Chulaimbo Sub-County Hospital with signs of uncomplicated malaria from the month of May 2015 to November 2015 were sampled for the present study.

### Inclusion criteria

We recruited children aged 6 months or above 60 months whose parents gave written informed content. In addition, the study population had the following common characteristics: they all visited the health facility with symptoms of malaria; had the body weight of ≥5 kg; confirmed mono-infection with *P. falciparum* and parasitaemia in the range of 2000–200 000 asexual parasites per microliter of blood, without symptoms of severe and complicated malaria, such as prostration, breathing difficulties, severe anaemia, convulsions and inability to drink or vomiting.

### Exclusion criteria

Among other things, patients below 6 months or above 60 months or whose body weight was <5 kg were not considered for participation. Similarly, those with a history of fever for more than 24 h with temperatures above 37.5 °C; multiple infections apart from malaria, such as evidence of liver infections; inability to take drugs orally, or was put under antimalarial medication within the previous 2 weeks were not considered potential participants. The same applied to potential participants whose parents/guardians remained uncooperative or failed to give a signed consent.

### Study design

This was a single-arm, prospective, *in vivo* study intended to assess the susceptibility of AL to drug resistance after previously being used to treat uncomplicated malaria. The assessment was conducted in accordance with the WHO guidelines [6].

### Sample size determination

The sample size determination was calculated using the formula *n* = *Z*^2^ *p*(1–*P*)/*e*^2^ as described by Lwanga and Lameshow [21], where:

*Z* = standard normal deviation of the required confidence

*n* = the desired sample size

*p* = proportion of the target population estimated to have suffered from and received treatment for Malaria. According to the Kenya Malaria Indicator Survey, the prevalence of malaria stands at 38% or 0.38 [22].

*e* = margin error or the desired precision

Therefore, upon substituting *Z* with 1.96, 0.38 for *P* and 0.1 for *e*, the minimum sample size was 1.96^2^ × 0.38 (1−0.38)/0.1^2^ = 90.

### Sample collection and testing procedures

Approximately, 0.05 ml of blood from a finger prick was collected. Thick and thin smears were prepared on two different slides. We examined slides stained with 10% Giemsa under the microscope to detect the presence of malaria parasites and estimated parasite densities. This was followed with a thin film blood slide stained with 3% Giemsa for parasite species determination and establishment of the presence of gametocytes. Parasitaemia was measured by counting the number of asexual parasites against 200 leucocytes in thick blood films. Parasite density per μl of blood was calculated by multiplying the total count by 40, assuming that 1 μl of blood had a mean count of 8000 leucocytes [23]. The blood slides were declared negative when the examination of 100 high-power fields did not show the existence of any malaria parasite. For quality control, each slide was re-examined by a second laboratory technologist who was blinded from the previous slide readings. The average of the two readings was used to determine the final parasitaemia. All the testing was done at the appointed health facility.

### Treatment, clinical monitoring and follow-up

Treatment with AL was done for 3 days in line with WHO weight-based regime [6]. A fixed-dose combination of 20 mg of artemether and 120 mg lumefantrine per tablet was administered, translating to one, two or three tablets per patient depending on individual weight. A full course of AL consisted of three tablets taken two times in a day (8 h apart on day 0, and 12 h apart on days 1 and 2) with no ‘gametocide drug’ added. After drug administration, patients were observed for 20 min to make sure they did not vomit. If vomiting occurred, a repeat dose was given. Any patient who persistently vomited was withdrawn and treated with parenteral artesunate or quinine according to the national guidelines for the management of severe malaria [24]. Besides, paracetamol was given to all patients with a body temperature of ≥38 °C. Patients were admitted at the health facilities for close monitoring and the drugs were administered orally at the health facility under direct observation of a nurse throughout the 3 days. The drug was crushed and mixed with a spoonful of porridge and little sugar to minimise vomiting and was given to each child to swallow. All drugs used in this study were provided by the Ministry of Health, Kenya.

Upon completion of the dose and recovery, the patients were allowed to go home. Follow-up visits were done on days 7, 14, 21 and 28 or at any time the patient felt unwell. Parents or guardians were informed and encouraged to bring their children to the clinic whenever they showed signs of being unwell and not necessarily wait for scheduled visits. For scheduled visits, parents who did not show up by mid-day of the appointed day were visited at home by a member of the study team and asked to report to the health facility. If a patient was not traced for scheduled follow-up, he/she was classified as lost to follow-up. During the scheduled visits, both clinical and parasitological assessments were performed.

### Treatment outcome classification

The classification of treatment outcome was either ETF, LCF, LPF or ACPR based on the WHO *in vivo* drug trial protocol of 2009 [6].

### Study outcome

#### Cure rate of AL

Of the 90 patients enrolled, 76 completed the follow-up at day 28 with ACPR. There was no treatment failure observed.

#### Parasite clearance rate

At recruitment, 19.7% (15/76) of the study participants were severely parasitaemic (>10 000 parasites/μl) while the rest 80.3% (61/76) were found moderately parasitaemic (1000–9999 parasites/μl). After medication, parasitaemia in the patients under observation was observed to decline as follows: 55.3% of parasitaemia cleared on day 1, 94.3% on day 2 while all the parasitaemia cleared on day 3.

#### Fever clearance rate

Febrile individuals, those with ≥37.5 axillary temperature, accounted for 71.1% (54/76) of the total study population on the day of recruitment. The number decreased to 28.9% (22/76) on day 1 followed by a total return to normal temperatures of 36.5 °C on day 2. There were not any febrile cases recorded on subsequent days.

#### Deoxyribonucleic acid (DNA) extraction

DNA was extracted from dried blood spot as described by Warhurst *et al*. 1991 [25]. Briefly, the Whatmann filter paper containing dried blood sample was shredded into several pieces and immersed into saponin-phosphate-buffered saline (PBS) overnight at 4 °C. The pieces were then washed with 1× PBS followed by incubation for approximately 30 min. During the washing, the brown solution from the tube was poured off and about 50 μl of 20% stock solution mixed with 150 μl of DNA set under running water followed by vigorous vortexing. The mixtures were vortexed vigorously and the tubes heated at 100 °C after which they were centrifuged at 10 000 ***g*** for 2 min. In the final step, the supernatant was transferred into another new tube, vortexed again before being transferred again to a new tube. The DNA product was then stored at −20 °C.

#### Amplification of Pf18sRNA gene (Pf3d7_1343700) for species confirmation

The primers pairs designed by Demas *et al*. [26] of 18R-18F were used; (5′-CTGAGTCGAATGAACTAGTCGCTAC-3′) and (5′-CCATTTTACTCGCAATAACGCT-3′). The PCR reaction included MgCl_2_, 400 nM, 200 nM of primers, 1 U of Taq Polymerase and 1 μl of DNA template. Amplification was done using PCR that included the initial denaturation at 94 °C for 3 min, followed by 30 cycles of denaturation at 94 °C for 1 min, annealing at 55 °C for 2 min, extension at 72 °C for 2 min and the final extension of 72 °C for 10 min. Finally, the reaction was held at 4 °C. The amplicons were analysed by electrophoresis in 1.5% molecular grade agarose gel and visualised by UV trans-illuminator. The expected band size of 800 bp was scored against 100 base pair DNA ladder.

### Data analysis

Data were entered in SPSS 17.0 version. Treatment outcome was classified according to clinical and parasitological responses using the WHO protocol (WHO, 2009). Differences between the lost respondents and those that completed the study were analysed using the *χ*^2^ test and two-sample *t* test. The time taken before parasite clearance after AL administration was determined using a Kaplan–Meier estimator (Fig. 4).

## Results

### Demographic characteristics

**Fig. 2.**
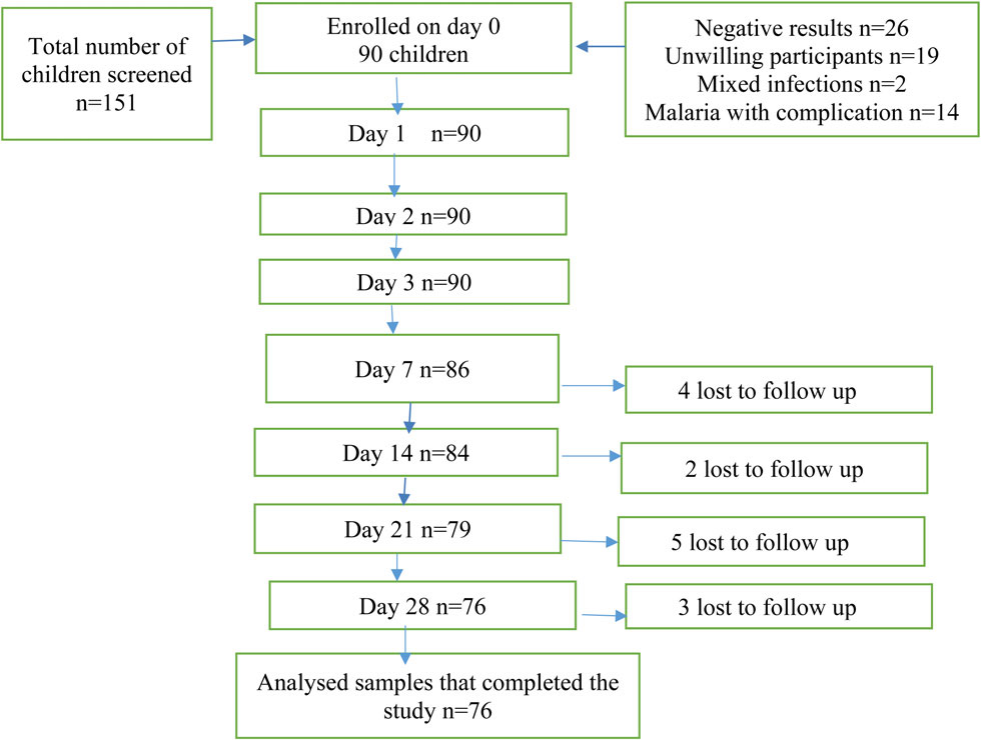
Figure showing the flow of patients during the screening, enrolment, and follow-up.

A total of 151 children were screened for eligibility to participate in the study from May to November 2015. Ninety of them fulfilled the inclusion criteria and were enrolled in the study. Of the 90 that were recruited, 76 (84.4%) completed the 28-day period while 14 (15.4%) were lost to follow-up (Fig. 2). Table 1 shows the profile of the children who participated in the study. All participants were confirmed cases of *P. falciparum* following a Pf18sRNA analysis. The response rate and the difference between those who got lost to follow-up and those stayed through were largely insignificant across analytical categories, as follows: gender (*χ*^2^ = 2.528, df = 1, *P* > 0.05); age (mean and s.d. of 32.1 and 10.7 for those who fell off and a mean and s.d. of 32.1 and 11.2 for those who completed). The same trend was observed for weight with a mean and s.d. of 15.6 and 3.2 for those that never completed and a mean and s.d. of 14.1 and 2.9 (*t*(87) = 1.722, *P* > 0.05) for those who completed.

**Table 1.** Profile of children on therapeutic in the study population

	Artemether–lumefantrine
Characteristics	*n* = 76
Male *n* (%)	43 (56.6%)
Female *n* (%)	33 (43.4%)
Median age in months (males)	30
Age range in months (males)	(12–56)
Median age in months (females)	30
Age range in months (females)	(15–58)
Temperature mean on day 0	38.1 °C s.d. ± 1.1
Temperature mean on day 3	36.5 °C s.d. ± 0.6
Parasitaemia (per μl) on day 0 geometric mean and s.d. respectively (95% CI 797.8–6220.7)	120 595.0 and 163 395.1
Day 2 mean and s.d.	24 and 170.9 (95% CI 15.0–63.3)
Range of parasites in the study sample	880–832 000

### Therapeutic efficacy outcomes

**Fig. 3.**
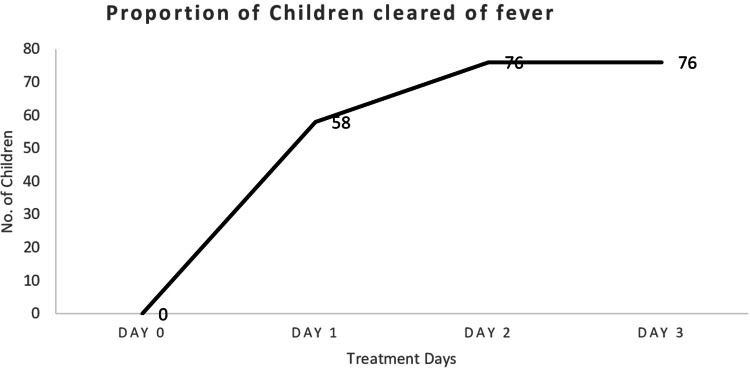
Fever clearance on the first three follow-up days.

**Fig. 4.**
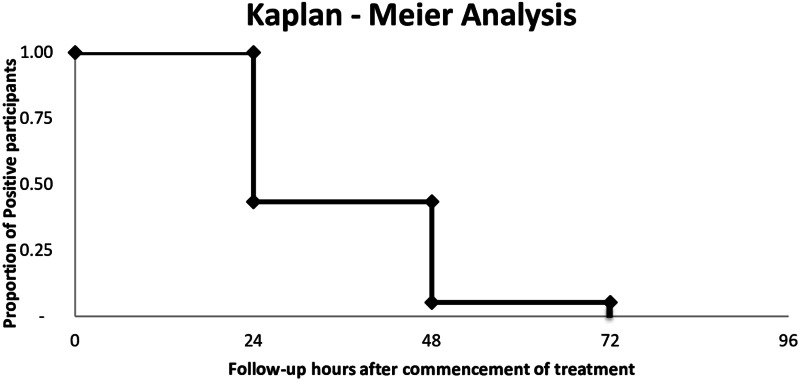
Kaplan–Meier plot of the time it took for the parasites to clear from participants' systems. The proportion of *P. falciparum*-positive children is shown on *y*-axis while the time of disappearance of the parasites is shown on the *x*-axis.

The therapeutic efficacy outcome is shown in Table 2. Within 48 h (2 days) of medication, 72 (94.2%) of the participants had cleared parasitaemia while three (5.8%) still had parasitaemia but which cleared on day 3. All fever cleared on day 2, as shown in Figure 3 below. Additionally, there was not a recrudescent infection observed up to the 28th day of the follow-up. There was also no serious adverse effect, such as vomiting, during the entire period of the follow-up. Equally, in Figure 4, below, the proportion of parasites cleared over time after the administration of AL is illustrated.

**Table 2.** Therapeutic efficacy of artemether–lumefantrine

Variables	Frequency (no. of individuals in each classification)
Parasitaemia on day 3	0 (0.0%)
ETF	0 (0.0%)
LCF	0 (0.0%)
LPF	0(0.0%)
ACPR	76(100%)
Initial no. of samples	90
Lost to follow-up on day 7	4
Lost to follow-up on day 14	2
Lost to follow-up on day 21	5
Lost to follow-up on day 28	3
Total baseline	76

## Discussion

A sudden and unprecedented upsurge of resistance to anti-malarial drugs can extensively reverse gains made in the fight and control of malaria [2, 27]. In this study, however, we observed a high cure rate and efficacy (100%ACPR) of AL, the approved drug for treating uncomplicated malaria Kenya over the past decade. There was a 100% adequate clinical and parasite clearance which compares well with studies in Papua New Guinea and Ethiopia that reported the efficacy rate of 97.8% and 98%, respectively [5, 28]. The related studies demonstrated an exemplary high curing rate of AL, especially in children aged <5 years [29]. Indeed, similar studies conducted in Tanzania, and in many other regions of Africa, replicated both the present and previous studies with high cure rates for AL, amid its prolonged use on the continent [30, 31]. The high parasite clearance rates may perhaps be described by the persistent act of artemether to clear parasites biomass leading to a rapid resolution of clinical manifestation. According to WHO, a suspected artemisinin resistance is when there is a delayed parasite clearance showing a slope half-life or a positive rate of 10% and over from within 5 h to day 3 of prescription [2]. The current study indicates the absence of resistance to artemisinin with a clearance of parasites within 24 h after AL. However, several studies undertaken earlier in Kenya as well as in Tanzania, Uganda, Somalia, Mali, Angola and several other African countries indicated that artemisinin resistance was already spreading in the African continent [24, 30, 32, 33]. The reporting of therapeutic failures calls for further investigation on molecular markers associated with AL, such as *Pfmdr1* gene and *K13 propeller gene*.

Although the current study did not include the examination of molecular markers associated with AL resistance, a number of related studies have done so with profound outcomes [34]. Several studies have linked codons F446I, N458Y, M476I, Y493H, R539T, P553L, R561H and C580Y of Pfk13 to delayed parasites clearance [34]. A study in Angola observed that the combination has been linked to a number of mutations in the *P. falciparum* kelch 13 resulting in a prolonged parasite clearance time, with C580Y being the most noted [35]. The kelch protein is believed to facilitate diverse cellular functions, including ubiquitin-regulated protein degradation and oxidative stress responses [36]. Another study observed that artemisinin tolerance *in vitro* was brought about by the *pfkelch13* M476I mutation while sustained parasite survival *ex vivo* was caused by Y493H, I543T, R539T and C580Y mutations while *in vivo* parasite, the half-life was amplified by Y493H, R539T, with the most prevalence being C580Y mutation [37, 38]. The prevalence of these mutant alleles of k13 has been shown to be involved in artemisinin resistance using genome-editing studies [36, 39]. Resistance to partner drugs has also been linked to polymorphism in certain genes. For example, increased copy number in *Pfmdr1* has been associated with increased inhibitory concentration of lumefantrine in *in vitro* studies [40]. In addition, reduced susceptibility to lumefantrine has been associated with both *pfmdr1* SNPs including N86 [41] as well as certain combination of SNPs, precisely the N86/184F/D1246 haplotype [42].

Among the artemisinin-based combination therapies, artesunate–amodiaquine, DHA/PPQ and AL are currently recommended ACTs for the treatment of uncomplicated *P. falciparum* malaria in endemic regions globally [2]. Even when studies continue to observe that these ACTs have maintained high efficacy (cure rate ≥95%) in many of these countries, despite their use for more than a decade [29], studies carried out in sections of Angola in 2013 and 2015 showed a lower efficacy (<90% cure rate) of AL [29]. In the latter studies, though, the administration of the evening dose was not supervised, hence no confirmation that this dose was consumed by the patient. The lower cure rate observed in the two regions of Angola in those 2 years may also have been as a result of sub-therapeutic doses of the AL. Indeed, the efficacious levels for AL reported in our study were also observed in northwest Ethiopia where there was the absence of ETF, confirming non-existence of possible artemisinin-resistant *P. falciparum* in the study area. Despite the study showing the absence of ETF with low recurrent malaria (1 LTF), the outcome of the Ethiopian study points to a highly therapeutic efficacy of both partners of AL [43].

Therefore, factors such as host immunity, nutritional factors, initial parasitaemia level, pharmacokinetics and pharmacodynamics may influence the therapeutic efficacy of a drug apart from inherent parasite susceptibility [44]. Any of the above parameters may provide a low efficacy of otherwise a highly efficacious drug. At the same time, resistant parasites may be cleared with the help of the immune system resulting in exaggerated efficacy of otherwise a less efficacious antimalarial drug [43]. For that reason, the unfortunate development and possible spread of artemisinin-resistance parasites in Southeast Asia and fluctuations in sensitivities to artemisinin partner drugs have raised concern globally [44].

Yet another study in Ethiopia recorded a paltry five treatment failures: 1 (1.1%) LTF and 4 (4.5%) were LPF compared with a majority 84 (94.4%) ACPR. The study authenticated the findings of the present study which indicate that the treatment of uncomplicated malaria using AL has a high clearance rate of a 100% ACPR. Indeed, the above cited studies have not only shown high efficacy of AL in the treatment of uncomplicated malaria but also correspond with findings from most east African countries. In addition, the ability of AL to rapidly eliminate *P. falciparum*, especially at its sexual stages, alongside the capacities of the partner drug on the gametocytes has been adequately demonstrated [45]. Parasitaemia has been associated with a degree of malaria severity and, thus, a significant consideration at the point of making decisions on the most appropriate type of treatment to be initiated. It is also an epidemiological inference parameter as it points towards the level of transmission in a specific area. The endemic level of malaria in the current study is described as holoendemic. Based on the health facility's official data, malaria incidence in the region is seasonal, subject to the quantity and length of rainfall.

There were no gametocytes detected from day 0 to 28 in the current study. A study in Thailand showed that when artemisinin derivatives were introduced as a component of the first-line treatment, there was a tremendous reduction in the incidence of clinical *P. falciparum* [45]. However, several studies have observed that ACT is linked to the decline in the transmission of malaria, which is due to the quick eradication of asexual parasites, and partly due to their properties on gametocytes [46]. Gametocytes clearance after treatment reduces the spread of malaria and neutralises the selection as well as the spread of resistance in malaria parasites. Additionally, ACT is highly effective against asexual stages and immature gametocytes [46]. Moreover, ACT is known for its rapid parasite clearance which is higher than other antimalarials such as quinine [43]. The antimalarial drug reduces the quantity of gametocytes (sexual stage of the parasites) which are responsible for the transmission of the infection to the vector, the Anopheles mosquito and the asexual parasites in a cycle that result in new gametocytes [47]. This should demonstrate how potent the antimalarial property of AL is in targeting the blood stage parasites.

This study observed that fever was linked to discomfort and was the key clinical manifestation in children with fever above 37.5 °C. The AL combination has been acknowledged as a rapid fever reducer and a long-acting drug to prevent recrudescence [44, 48, 49]. Data obtained from South-east Asian countries such as Thailand and Cambodia including some African countries indicated that there is delayed fever clearance after AL treatment [49]. The key concern for ACTs in Africa, particularly in areas of severe transmission, is the slow pace of clearance of the parasite thus aiding the development of resistant strains. There is, therefore, a compelling need for continuous surveillance of the drug's efficacy.

## Conclusion

This study concludes that AL continues to be effective against uncomplicated malaria caused by *P. falciparum* in Kenya and beyond. It is observed that the fact that AL has been reliably in use for a decade explains why it is the best-choice medication to treat uncomplicated malaria caused by *P. falciparum*. Be that as it may, intensive and ‘regular surveillance of ACT partner drugs needs be conducted’ to not only ensure early detection of resistance to *P. falciparum* but also guarantee informed decisions by policy makers on matters of malaria treatment.

### Limitation of the study

In this study, only those participants that remained under observation for the period of the study (28 days) were relied on for the conclusions presented in this study. It was therefore difficult to decisively rule out treatment failures that may not have been detected or accounted for, especially among participants that got lost to follow-up. We indeed attest that variables used (age, sex and weight) may not adequately reveal the whole extent of the impact of failure to account for the lost participants. Nevertheless, much as the sample size was small, including those lost to follow-up, the study outcome may reliably be generalised to related contexts.

## Data Availability

The raw data are available on request by the editor of the publishing journal.
